# Baseline Impaired Insight Predicts Longitudinal Brain Atrophy in Alzheimer's Disease and Related Cognitive States: A 30‐Month Cohort Study From the ADNI Dataset

**DOI:** 10.1002/brb3.70893

**Published:** 2025-09-30

**Authors:** Jacob Wilkins, Carlos Muñoz Neira, Li Su, Alzheimer's Disease Neuroimaging Initiative

**Affiliations:** ^1^ Sheffield Institute for Translational Neuroscience (SITraN), Division of Neuroscience, School of Medicine and Population Health, Faculty of Health University of Sheffield Sheffield UK; ^2^ Insigneo Institute for In Silico Medicine, School of Medicine and Population Health University of Sheffield Sheffield UK; ^3^ Department of Psychiatry, School of Clinical Medicine University of Cambridge Cambridge UK

## Abstract

**Background:**

Impaired insight can be understood clinically as a loss of ability to appropriately recognize one's own disease status. Investigating insight in Alzheimer's disease (AD) and its relation to longitudinal changes in brain structure is important to understand the disease progression.

**Objective:**

To examine how the character of insight changes with disease stage and assess whether baseline levels of impaired insight can predict rate of brain atrophy across a period of 30 months in a cohort of subjects consisting of subjective memory complaint (SMC), mild cognitive impairment (MCI), AD, and cognitively normal (CN) controls.

**Methods:**

Data from 794 eligible participants were extracted from the Alzheimer's Disease Neuroimaging Initiative (ADNI) dataset. Insight levels were estimated by the Measurement of Everyday Cognition (ECog). Impairment was further categorized into overestimation or underestimation of ability. Brain atrophy rates were estimated by measuring change in gray matter volume within 30 months.

**Results:**

Overestimating ability was significantly correlated with increased whole‐brain atrophy rates (*p* < 0.001) independent of general cognitive decline. Overestimation of ability exhibited significant correlations with increased atrophy in specific regions of the brain including the medial temporal lobe, fusiform gyrus, and hippocampus.

**Discussion:**

The present results suggest a statistically significant correlation between overestimation of ability and increased rates of subsequent brain atrophy. This is particularly notable in regions of the brain such as the hippocampus. However, further study into the phenomenon of insight and its progression over the disease course is required before its potential clinical utility can be fully assessed.

## Graphical abstract

This research explored whether the psychological marker of insight in participants with Alzheimer's disease and other cognitive states was correlated with brain atrophy on MRI over a 30‐month period. The research found that overestimation of ability is correlated with increased rates of brain atrophy in this ADNI cohort.

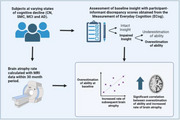

**Abbreviations**
AD: Alzheimer's disease
ADNI: Alzheimer's Disease Neuroimaging Initiative
ANOVA: analysis of variance
CN: cognitively normal
ECog: Everyday Cognition Scale
EMCI: early mild cognitive impairment
FDR: false discovery rate
ICV: total intracranial volume
LMCI: late mild cognitive impairment
MCI: mild cognitive impairment
MMSE: Mini‐Mental State Examination
MoCA: Montreal Cognitive Assessment
MRI: magnetic resonance imaging
MTL: medial temporal lobe
NoV: number of visits
SMC: subjective memory complaint
YoE: years of education

## 1 Introduction

Dementia is a brain disorder characterized by the presence of cognitive decline and symptoms that decrease one's ability to function independently including memory loss, impaired executive functioning, language impairment, and apraxia (Duong et al. 2017). A commonly seen, although largely unexplored symptom, is a lack of insight into diagnosis or symptoms, which appears to occur in anywhere between 20% and 80% of mild dementia cases (Wilson et al. 2016). Its prevalence and severity rise with the progression of the disease (Cacciamani et al. 2021; Starkstein 2014).

Levels of insight can vary prior to a clinical dementia presentation, with specific subdomains such as memory being affected 2–3 years prior, presenting at a similar time to early signs of regional brain atrophy (Whitwell 2010; Wilson et al. 2015). It is also found that impaired self‐perception in mild cognitive impairment (MCI), a prodromal stage of dementia, is predictive of the likelihood of progression to dementia in a 2‐year time frame (Therriault et al. 2018). Investigating altered insight in Alzheimer's disease (AD), which is the most common cause of dementia, can be particularly relevant considering its prevalence and clinical significance (about 75% of patients with AD experience severely impaired insight) (Wedderburn et al. 2008; Koch and Iliffe 2010; van Vliet et al. 2012).

Interestingly, brain regions affected first in AD such as the medial temporal lobe (MTL) have been associated with global impaired insight in neurodegenerative diseases (Chavoix and Insausti 2017) and specifically in AD (Tondelli et al. 2018). A faster decline of MTL volume has been shown in patients with AD compared to controls (Smith 2002) and MTL atrophy has also been shown to predict progression from MCI to AD dementia (Visser et al. 2002; Whitwell et al. 2007). Other studies have found that brain regions, including the fusiform, hippocampal, and inferior temporal areas, are the strongest predictors of progression to dementia (Kwak et al. 2022); however, it is unclear how these areas relate to impaired insight.

Given the evidence from the literature, we argue that clinical assessment of altered insight may be useful in identifying those who are showing early signs of dementia. Likewise, impaired insight may turn into a novel predictive neuropsychological marker for the progression of AD. Based on the Alzheimer's Disease Neuroimaging Initiative (ADNI) database (https://adni.loni.usc.edu), this study is primarily aimed at investigating whether baseline levels of insight predict the longitudinal brain atrophy rates across the pathological timeline from healthy cognition and subjective memory complaint (SMC) to MCI and AD over a 30‐month period. We will further explore the associations of specific subdomains of insight with key regions of the brain associated with AD pathology. To our best knowledge, this is the first study that examines variations of insight and brain atrophy rates over time across the continuum ranging from preserved cognition to clinical dementia.

## 2 Methods

### 2.1 Data Collection

Data used in the preparation of this article were obtained from the ADNI, which was launched in 2003 as a public‐private partnership, led by Principal Investigator Michael W. Weiner, MD. The primary goal of ADNI has been to test whether serial magnetic resonance imaging (MRI), positron emission tomography (PET), other biological markers, and clinical and neuropsychological assessment can be combined to measure the progression of MCI and early AD.

### 2.2 Subjects

Participants taken from the ADNI1, ADNI2, ADNI3, and ADNIGO studies were grouped across five diagnostic subsamples: cognitively normal (CN), SMC, early MCI (EMCI), late MCI (LMCI), and AD. Subgroups were categorized according to predefined criteria (Aisen et al. 2015).

To be included in this analysis, all participants were required to have completed both participant's and informant's versions of the Everyday Cognition Scale (ECog) (Tomaszewski Farias et al. 2011) at their baseline visit so that discrepancy scores (an index of insight) could be calculated. Participants had to have undergone MRI brain scan at the baseline visit and at least one follow‐up visit so that longitudinal brain volume change could be calculated and compared. For participants with only two visits, only those with visits at least 12 months apart were eligible to obtain a reliable estimate. The cutoff for follow‐up visits included in this study was month 30, as this was the last month in which there was data available for any AD participants, ensuring the total number of data points is comparable between groups. The ADNI database was filtered subject to these criteria resulting in a sample population of 817 eligible participants. Global cognitive efficiency of subgroups was evaluated with the Mini‐Mental State Examination (MMSE) and the Montreal Cognitive Assessment (MoCA) (Folstein et al. 1975; Nasreddine et al. 2005).

### 2.3 Insight Assessment

To assess baseline levels of insight, participant's and an informant's responses to the ECog were used. The ECog is a 39‐item questionnaire covering six cognitive subdomains: language, memory, divided attention, visuospatial ability, planning, and organization (Tomaszewski Farias et al. 2011).

The informant's responses were used as a disease‐independent reference to compare with the assessment of the participants’ ability, and therefore the discrepancy scores between participants’ and informants’ answers were used as proxy measures of insight. To create these measures, the participants’ scores were subtracted from the informants’ scores. Intact insight will have a discrepancy score around zero. A negative discrepancy score or an underestimation means someone has a lower self‐perception of their own ability. A positive score is referred to as overestimation. As well as calculating “total insight,” the ECog questionnaire was broken down into six cognitive subdomains (memory, language, visuospatial, planning, organizational skills, and divided attention) so insight into specific domains was also calculated.

### 2.4 MRI Acquisition and Analysis

High‐resolution 3 Tesla MRI brain scans at baseline and subsequent visits were collected following the protocols outlined by ADNI. Image derived metrics were available for download from ADNI. T1‐weighted MRI data taken from 3 Tesla high‐resolution brain scans were analyzed by ADNI using the FreeSurfer Segmentation tool (http://surfer.nmr.mgh.harvard.edu/, version 5.1 for ADNI1, 2, GO and version 6.0 for ADNI3) with procedures primarily outlined by Fischl et al. (2002).

**FIGURE 1:**
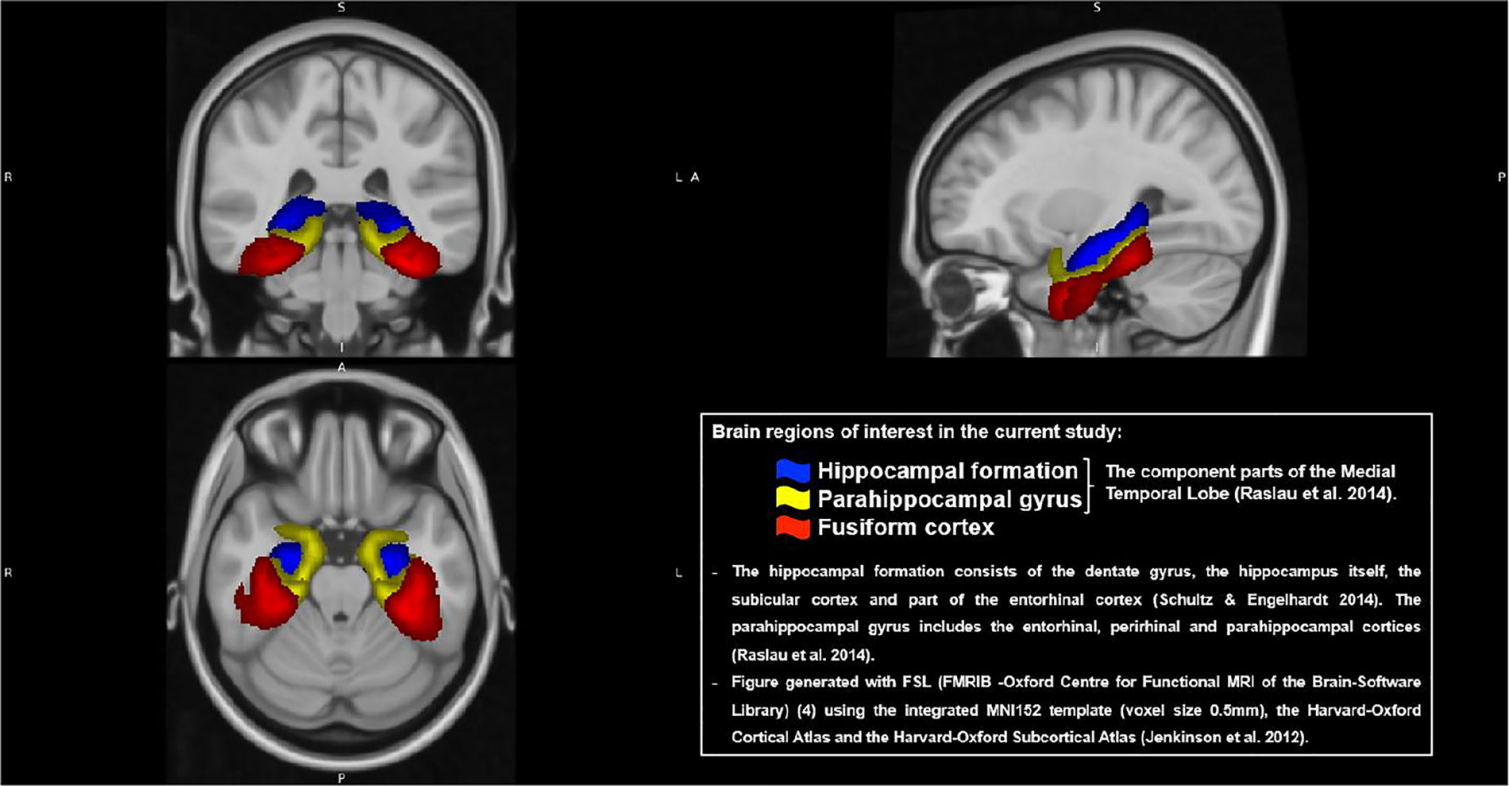
Brain mapping of regions included in the current study.

Whole‐brain and regional gray matter volumes (from hippocampi, fusiform gyri, entorhinal cortices, and MTLs) that can predict progression to dementia or underpin early AD pathology based on the literature were included (Kwak et al. 2022; Planche et al. 2022; Rao et al. 2022). For the brain subdivisions, this is the total of the left and right regions in each subject. To minimize variation between individual differences in total intracranial volume (ICV), the ratio of the participants’ brain volume in question to the ICV was calculated for each visit. A summary of the subregions included in the study is seen in Figure 1.

### 2.5 Annualized Brain Atrophy Rate

For the whole brain and each brain region, the gray matter volume to ICV ratio was used as the dependent variables and the months from baseline as the independent variable. Slopes were calculated employing a least squares regression assuming a linear relationship between time and brain volume change. These data were converted to an annualized percentage decrease as a proportion of the ICV for use in data visualization, which is referred to as rate of atrophy.

Slopes were also calculated for the participants’ ICV changes over time. This was done so participants with large changes in ICV volume over time could be identified and excluded, as it was assumed that ICV would not have large deviations between visits. Participants whose ICV slope was three times the interquartile range were counted as extreme outliers and were removed, as they were likely to heavily skew the data.

### 2.6 Statistical Procedures

Statistical analysis was performed in Jeffreys's Amazing Statistics Program JASP Version 0.17.2. The statistical significance for all analyses was set at *p* < 0.05, and where multiple comparisons were made, a false discovery rate (FDR) correction was applied using the Benjamini–Hochberg method (Benjamini and Hochberg 1995).

A comparison of the distribution of possible confounding variables in the association between insight and brain volumes between diagnostic groups was completed. Examination of potential differences between groups was performed using standard statistical testing dependent on the normality of the demographic variable, assessed via Shapiro–Wilk tests. As gender is a categorical variable, comparisons between groups were completed with a chi‐squared test.

The distribution of insight scores in different diagnostic groups was compared through visual inspection using histogram plots to assess the character of insight depending on the diagnosis. Complementary, insight scores and atrophy rates were assessed statistically with Shapiro–Wilk testing to determine whether parametric or nonparametric statistical correlation methods should be used. Correlations devoted to evaluating insight's predictive value in brain changes across time were controlled for confounding variables including age, years of education (YoE), sex, number of visits (NoV), and degree of cognitive efficiency as discussed in the next section.

### 2.7 Confounding Variables

The correlation between subdomains of insight and atrophy rates needed to be corrected for certain confounding variables, given that brain atrophy occurs with natural aging even in the CN elderly (Erten‐Lyons et al. 2013). Sex‐specific differences are also present within AD pathology (Kadlecova et al. 2023). Concerning YoE, although it has been reported that this factor does not shape cognitive decline (Berggren et al. 2018), the positive influence of literacy on neuropsychological tasks is widely known (Lezak et al. 2004), and therefore, participants with a greater number of YoE may exhibit higher levels of insight as a result. In addition, increased levels of education have been suggested to be protective against AD‐related brain pathology, further highlighting the need to control this variable (Zhu et al. 2021). Selection criteria used here allowed for people with differing NoV for MRI scans. To account for this variability, the NoV was used as a covariate. To assess insight's use in prognosis, it needed to be separated from the potential covariate of baseline burdens and severity of AD which could be measured using MoCA or MMSE; however, the MoCA was selected here because this tool has been shown to be more sensitive for patients with MCI (Nasreddine et al. 2005), more suitable than the MMSE to detect cognitive decline (Pinto et al. 2018), and a better measure of global cognition (Jia et al. 2021).

## 3 Results

### 3.1 Participant Demographics

After filtering for the required variables in the ADNI database and checking for extreme outliers in the ICV slope distribution, 23 participants were excluded, leaving a total sample size of 794. This was distributed across five groups of differing diagnoses at baseline: CN (*n* = 176), SMC (*n* = 150), EMCI (*n* = 245), LMCI (*n* = 148), and AD (*n* = 75).

For the demographic variables: age, YoE, gender, MoCA, and NoV, Shapiro–Wilk testing was employed to assess whether parametric or nonparametric testing was required for differences between groups. Testing suggested that age followed a normal distribution, but the other variables did not. Significant differences were found in all variables: age (*p* = 0.034), NoV (*p* < 0.001), YoE (*p* = 0.048), gender (*x* ^2^ = 19.915, *p* < 0.001), and MoCA (*p* < 0.001), suggesting that these variables would all need to be controlled for throughout further statistical analyses. Demographic information is detailed in Table 1.

**TABLE 1:** Demographic data of the study population.

	Whole cohort	CN	SMC	EMCI	LMCI	AD	Group differences
Number of participants	794	176	150	245	148	75	
Mean age	71.487	72.253	70.953	70.866	71.206	73.265	*p* = 0.034
	(SD = 6.821)	(SD = 6.157)	(SD = 5.564)	(SD = 6.948)	(SD = 7.680)	(SD = 7.993)	
Mean years of education	16.486	16.898	16.48	16.433	16.405	15.867	*p* = 0.048
	(SD = 2.517)	(SD = 2.383)	(SD = 2.495)	(SD = 2.588)	(SD = 2.592)	(SD = 2.379)	
Mean baseline MoCA	24.018	26.171	26.041	24.189	22.377	17.394	*p* ≤ 0.001
	(SD = 3.852)	(SD = 2.278)	(SD = 2.744)	(SD = 2.773)	(SD = 3.167)	(SD = 4.400)	
Mean number of clinic visits	3.476	3.659	2.46	3.971	3.628	3.16	*p* ≤ 0.001
	(SD = 1.168)	(SD = 1.175)	(SD = 0.701)	(SD = 1.053)	(SD = 1.139)	(SD = 1.001)	
Gender (M = male, F = female)	M = 399	M = 82	M = 54	M = 137	M = 85	M = 41	*x* ^2^ = 19.915
	F = 395	F = 94	F = 96	F = 108	F = 63	F = 34	*p* ≤ 0.001

*Note*: Showing mean age, years of education, baseline MoCA, number of clinic visits, and gender. Analysis of variance (ANOVA) (age), Kruskal–Wallis (years of education, baseline MoCA, and number of clinic visits), and chi‐squared (gender) tests for group differences (*p* < 0.05).

### 3.2 Insight Levels by Diagnostic Group

**FIGURE 2:**
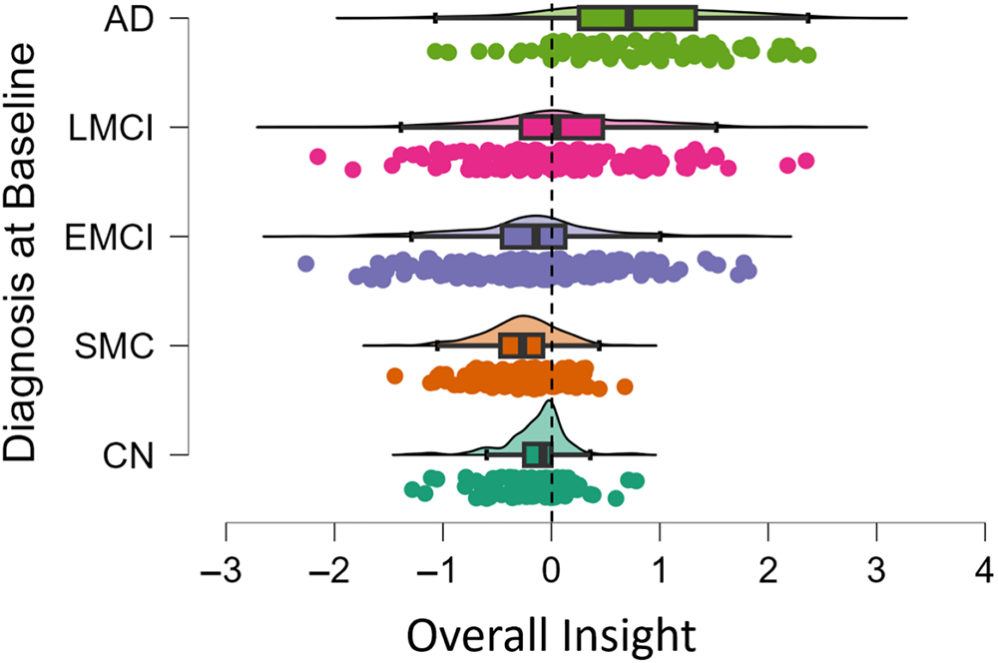
A raincloud plot of the distribution of (over‐ or underestimation of) insight scores by diagnostic group.

The type of overall insight loss was characterized by separating underestimation from overestimation. Statistically significant differences were seen between groups (*p* < 0.001), with post hoc comparisons detailed fully in Table S1. The SMC, CN, and EMCI groups underestimated their abilities on average, with SMC to the highest degree (*m* = −0.281, SD = 0.339), followed by EMCI (*m* = −0.175, SD = 0.649) and CN (*m* = −0.137, SD = 0.288). The LMCI and AD groups both overestimated their ability on average, but LMCI (*m* = 0.099, SD = 0.734) to a lesser extent than the AD group (*m* = 0.752, SD = 0.799). The distribution of insight character in different groups was visualized in Figure 2.

### 3.3 Brain Atrophy Rates

**FIGURE 3:**
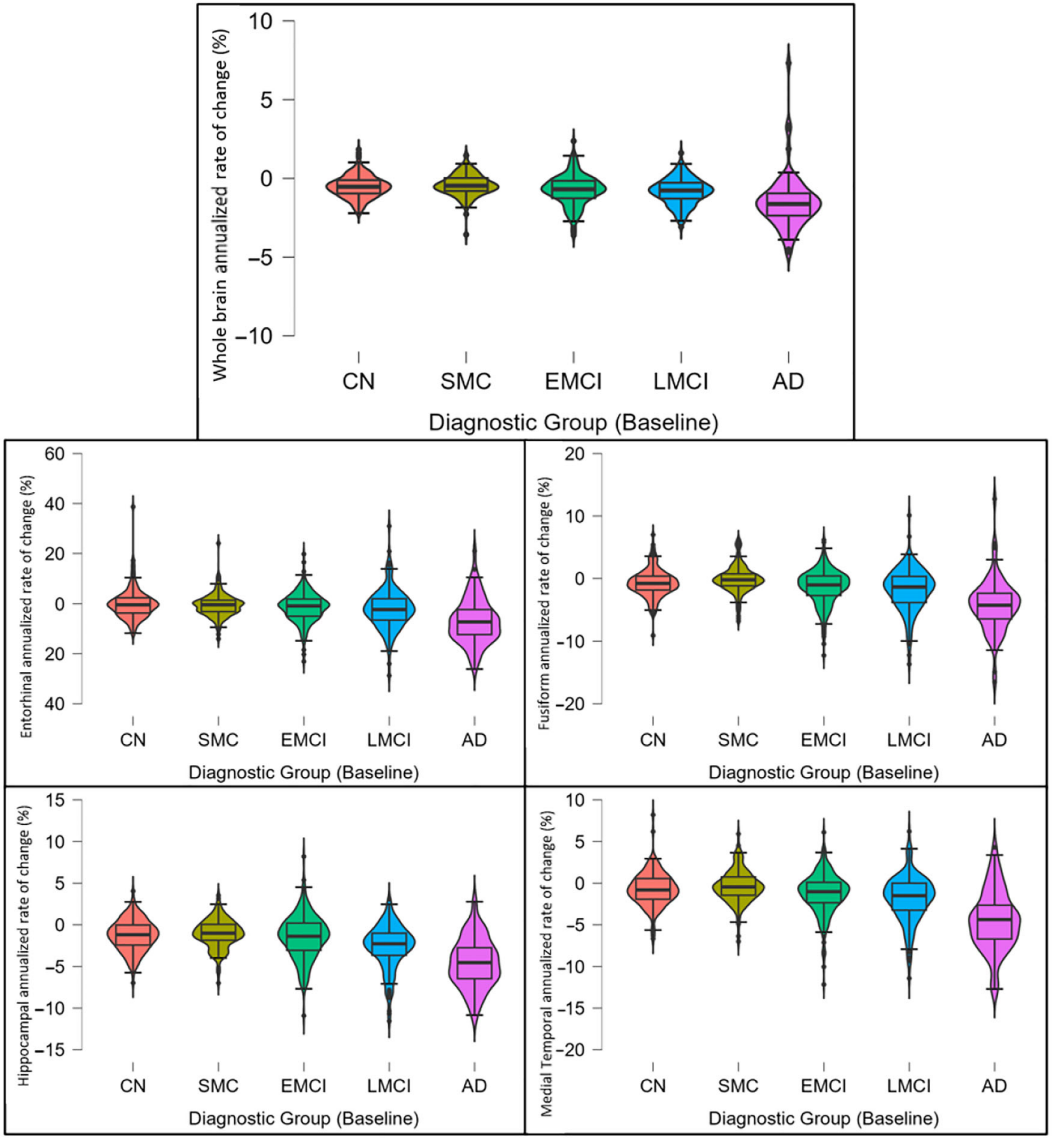
Violin plots showing the distributions of whole‐brain and specific region annualized atrophy rates by diagnostic group.

For the whole brain and all subregions of interest, a negative mean annualized percentage change as a proportion of total ICV was observed in all groups (means displayed in Table 2). The AD group showed the greatest mean brain atrophy rates in the whole brain and regional analysis. This was followed by LMCI, EMCI, CN, and finally the SMC group, which showed the lowest atrophy rates (Figure 3). These differences were found to be statistically significant between the groups (*p* < 0.001), and the full results of post hoc comparisons can be found in Table S2.

**TABLE 2:** Yearly atrophy rate of brain regions, split by diagnostic group.

		CN	SMC	EMCI	LMCI	AD
Whole‐brain yearly atrophy rate	Mean (%)	−0.519	−0.425	−0.756	−0.84	−1.492
	Standard deviation	0.717	0.694	0.969	0.831	1.752
Entorhinal yearly atrophy rate	Mean (%)	−0.053	−0.621	−1.431	−2.109	−6.929
	Standard deviation	5.973	4.625	5.718	8.052	8.97
Fusiform yearly atrophy rate	Mean (%)	−0.708	−0.097	−1.344	−1.773	−4.196
	Standard deviation	2.225	2.213	2.753	3.367	4.29
Hippocampus yearly atrophy rate	Mean (%)	−1.273	−1.062	−1.479	−2.661	−4.602
	Standard deviation	1.841	1.708	2.585	2.661	2.935
Medial temporal yearly atrophy rate	Mean (%)	−0.721	−0.397	−1.257	−1.879	−4.505
	Standard deviation	1.986	1.931	2.44	2.799	3.577

For the AD group, the entorhinal cortex showed the greatest atrophy rate in comparison to other brain regions, whereas the hippocampus had the highest rate of atrophy in the other groups (Table 2).

### 3.4 The Correlation Between Overall Insight and Rate of Whole Brain Changes

This analysis was undertaken to assess overall insight's effect on whole‐brain atrophy rates. Shapiro–Wilk testing of overall insight and whole‐brain atrophy rates showed that both variables followed a nonparametric distribution; therefore, the Spearman's rank was employed to assess the correlation between the variables. After accounting for the covariates: age, YoE, gender, baseline MoCA scores, and NoV, the Spearman's rank showed a significant negative correlation between the whole‐brain atrophy rate and overall insight (*r*_s_ = −0.128, *p* < 0.001), meaning that overestimation of overall ability was significantly correlated to increased whole‐brain atrophy rate.

### 3.5 The Correlation Between Overestimation and Rate of Regional Brain Changes

This analysis was conducted to evaluate the association between subdomains of insight and brain regions of interest that were selected based on the literature for early AD pathology. This was completed with a partial Spearman's rank that was controlled for the covariates: age, YoE, gender, MoCA scores, and NoV. For all the following statistically significant results, there was a negative correlation between insight domains and yearly atrophy rate across specific brain areas. This means that in all significant correlations, an overestimation of ability was correlated to increased rates of regional brain atrophy.

**FIGURE 4:**
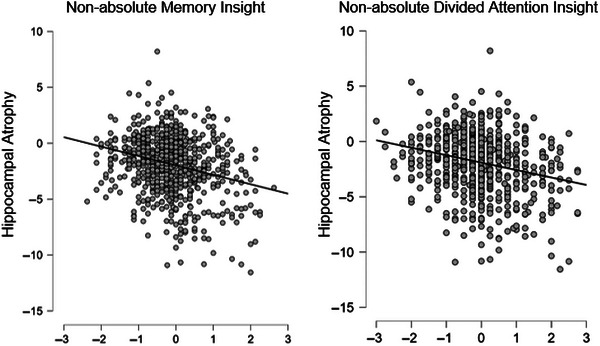
Scatter plots showing overestimation in memory and divided attention with increased rates of annualized hippocampal atrophy.

A summary of Spearman's rho, *p* values, and FDR corrections can be found in Table 3 for all partial correlations. Overestimation of visual‐spatial, organizational, and divided attention abilities was significantly correlated with an increase in rate of atrophy in all brain regions studied. Overestimation of memory, language, and planning ability was significantly correlated with an increase of fusiform, hippocampal, and medial temporal atrophy, but not entorhinal atrophy. Although some significant findings have modest effects, the strongest associations were hippocampal change with overestimation of divided attention ability (*r*_s_ = −0.167, FDR *p* = 0.002) and overestimation of memory ability (*r*_s_ = −0.165, FDR *p* = 0.002). Such relationships were visualized in the scatter plots in Figure 4.

**TABLE 3:** Results of Spearman's rho correlations between insight (overestimation/underestimation) split into cognitive subdomains and subregions of the brain affected in early Alzheimer's disease pathology.

		Entorhinal atrophy	Fusiform atrophy	Hippocampus atrophy	Medial temporal atrophy
Memory insight	Spearman's rho	−0.035	−0.148^***^	−0.165^***^	−0.124^***^
	*p* value	0.33	< 0.001	< 0.001	< 0.001
	FDR correction	0.33	0.002^**^	0.002^**^	0.002^**^
Language insight	Spearman's rho	−0.043	−0.134^***^	−0.134^***^	−0.123^***^
	*p* value	0.231	< 0.001	< 0.001	< 0.001
	FDR correction	0.241	0.002^**^	0.002^**^	0.002^**^
Visuospatial insight	Spearman's rho	−0.073^*^	−0.124^***^	−0.103^**^	−0.104^**^
	*p* value	0.043	< 0.001	0.004	0.004
	FDR correction	0.0491^*^	0.002^**^	0.0064^**^	0.0064^**^
Planning insight	Spearman's rho	−0.049	−0.109^**^	−0.118^**^	−0.091^*^
	*p* value	0.176	0.002	0.001	0.012
	FDR correction	0.192	0.00369^**^	0.002^**^	0.016^*^
Organizational insight	Spearman's rho	−0.075^*^	−0.122^***^	−0.148^***^	−0.1^**^
	*p* value	0.042	< 0.001	< 0.001	0.007
	FDR correction	0.0491^*^	0.002^**^	0.002^**^	0.00988^**^
Divided attention insight	Spearman's rho	−0.076^*^	−0.14^***^	−0.167^***^	−0.098^**^
	*p* value	0.036	< 0.001	< 0.001	0.007
	FDR correction	0.0455^*^	0.002^**^	0.002^**^	0.00988^**^

*Note*: Controlled for the covariates of age, gender, number of visits, years of education, and baseline MoCA score. Benjamini–Hochberg false discovery rate (FDR) correction of *p* values is applied.

^*^Significant after FDR correction, *p* < 0.05; ^**^ *p* < 0.01; and ^***^ *p* < 0.001.

## 4 Discussion

The study has found evidence to support the hypothesis that insight and its subdomains are significantly correlated to rates of brain atrophy. It also adds weight to the idea that insight is a phenomenon that is formed of multiple parts of the brain, and as such, specific region brain atrophy rates are variably associated with separate cognitive subdomains of insight. However, the atrophy rates of brain regions found to be significantly associated with insight tended to be associated with multiple, if not all, subdomains. This implies that self‐perceptive ability is tied to core regions of the brain, regardless of ability studied.

The literature regarding insight and its measurement in MCI and AD is inconsistent in its definition and methodology, making comparisons between studies difficult. However, in accordance with the current study, a recent meta‐analysis and systematic review of awareness of cognitive deficits (analogous to insight) found that MCI patients had significantly poorer awareness than controls, and mild AD patients had significantly worse awareness than MCI patients (Cacciamani et al. 2021).

Further categorizing impaired insight into an overestimation or an underestimation of ability suggested that, on average, the SMC, CN, and EMCI groups underestimated their ability, whereas at the later stages of the disease, LMCI and AD overestimated their ability. There is a growing suggestion that SMC may be thought of as a potential “pre‐MCI” stage and an important part of the prodromal stage of AD (Warren et al. 2022). A meta‐analysis of the progression of SMC to MCI and AD found that older people with SMC were twice as likely to develop dementia than those without SMC, with 6.6% of SMC patients progressing to MCI yearly, strengthening the argument that it is a possible stage before MCI (Mitchell et al. 2014). Although not statistically significant, we found a deepened underestimation of ability in SMC compared with CN. These findings highlight a further need to explore how the symptom of insight impairment changes longitudinally through the prodromal stages of AD.

Fusiform and medial temporal atrophy have been shown to occur in AD up to 3 years prior to clinical diagnosis, accelerating with the disease stage from cognitively normal to AD (Whitwell 2010; de Flores et al. 2022). This is supported by the current study which showed a significantly increased atrophy for these areas in the MCI and AD groups when compared to the CN, with atrophy rates also found to progressively increase with the stage of disease, with the exception of SMC. More research into SMC as a possible prodromal dementia stage is needed before concrete conclusions are made, given that atrophy rates were found to be slower than CN. One possible suggestion is that SMC may exhibit compensatory changes before structural abnormalities develop, which may explain the decreased atrophy rates seen here (Kawagoe et al. 2019). Given the modest brain atrophy in SMC, investigating neuropsychological changes including insight may shine some light on early detection of AD at the SMC stage.

Insight as a phenomenon has been previously associated with certain areas of the brain, such as the MTL, and impairment of this capability has been linked with an increased progression from MCI to dementia (Wilson et al. 2015; Therriault et al. 2018). However, the association of insight to rates of brain atrophy was unexplored in the past. The current study showed significant associations between baselines impaired insight and rate of whole‐brain change, even after accounting for several covariates and particularly the baseline MoCA scores, implying that this association is independent of the disease severity. The increased rate of atrophy suggests that those who demonstrate more impaired insight at any stage of AD are likely to be followed by a more aggressive disease course.

Although the pathogenicity of overestimation instead of “nondirectional awareness” of ability is largely unexplored, one study suggested that overestimation of functioning was more related to neuronal circuit dysfunction than that of underestimation in neurodegenerative diseases (Shany‐Ur et al. 2014). Furthermore, it has been found that overestimation of performance in a memory task was related to lower cortical thickness in AD‐vulnerable regions and executive decline (Sánchez‐Benavides et al. 2022). This supports the suggestion that overestimation of ability is a stronger marker of pathogenicity in AD than underestimation in terms of character of impaired insight. This is highlighted further by the significant correlations between overestimation of all cognitive subdomains and increased rates of atrophy of the MTL and hippocampus, but interestingly also the fusiform gyrus. The MTL and hippocampus have been highlighted as key regions linked with self‐perceptive ability and therefore crucial for maintaining insight into one's abilities (Hallam et al. 2020). The fusiform's association with insight has a basis within the literature, with studies suggesting that the fusiform gyrus, specifically the left, is important in visuomotor and perceptive self‐awareness (Li et al. 2021; Tacikowski et al. 2017).

Previous literature has suggested that atrophy of the MTL is a good predictor of progression from MCI to AD (DeCarli 2007); however, more recent studies have identified the hippocampus and fusiform region atrophy as stronger predictors of AD progression (Kwak et al. 2022). Given the significant association between the rate of atrophy in these areas and overestimation of ability, it may be possible to use a noninvasive neuropsychological assessment of insight as a proxy to estimate atrophy rates, and therefore the risk of disease progression. Although significant, the associations between levels of insight and brain atrophy rate are weak, suggesting a limited use in their predictive ability. Nevertheless, given AD is a multifactorial disorder, impaired insight may still have a use as a constituent factor in building a predictive model of both progression to AD and likely severity of disease course.

### 4.1 Limitations and Future Directions

Although the sample size is a strength of this study, this could only be achieved by including participants from multiple ADNI studies. While the ADNI database attempts to maintain strong continuity in harmonized procedures, slight differences are likely to be present, which may affect the reliability of the results.

In addition, insight was only considered at baseline visit, and while comparison was available between participants at different stages of disease progression, longitudinal insight changes of an individual were not studied. A follow‐up study of individual participants' insight changes longitudinally will allow for better characterization of insight over the course of a progression from CN to dementia.

There is also a possible selection bias for those who have no objective memory deficits but have self‐reported a memory complaint being more likely to underestimate ability (Chao et al. 2020). Further study into whether this group represents a prodromal stage of dementia or is a normal subpopulation of the CN is required.

Although many regions indicated in early disease were studied, other areas that have been reported to be important in self‐perceptive ability, such as the inferior frontal gyrus and the anterior cingulate cortex, were neglected in the study (Hallam et al. 2020). This may lead to under‐appreciating the association of the rate of brain atrophy with potentially important predictors. A comprehensive study of insight's relationship to atrophy rates in all the brain's constituent subregions could provide a more comprehensive view of insight's neurological basis and highlight more predictors of brain atrophy.

In addition, more extensive profiling of one's insight, particularly with a focus on overestimation in domains more strongly associated with brain atrophy, may help provide a stronger predictive picture of disease course.

Recent literature has highlighted the growing role of explainable artificial intelligence in AD, particularly in the field of neuroimaging (Taiyeb Khosroshahi et al. 2025; Viswan et al. 2023). Using models to further subcategorize brain regions associated with insight may highlight stronger markers to use in AD prognostics. In addition, research into whether artificial intelligence models could be trained on a range of noninvasive markers to be used in the prediction of AD disease course would potentially create clinical applications of this area of research.

## 5 Conclusion

AD is a growing issue, and better diagnostic and prognostic markers are required. Given brain changes have been shown to occur multiple years before diagnosis, early‐stage markers are likely to help with this challenge (Whitwell 2010). The current study provides early evidence that insight follows a dynamic path through the dementia disease course and that the neuropsychological measure—insight may have a role to play in predicting disease progression. This study highlights the potential usefulness of research into the phenomenon of insight and its relationship to dementia.

## Author Contributions

**Jacob Wilkins**: writing – original draft, investigation, conceptualization, methodology, visualization, writing – review and editing, software, formal analysis, data curation, resources. **Carlos Muñoz Neira**: investigation, writing – review and editing, software, supervision, conceptualization. **Li Su**: conceptualization, funding acquisition, writing – review and editing, project administration, supervision.

## Ethics Statement

The current study used previously anonymized data from the ADNI protocol. The ADNI protocol was carried out with the ethical standards of and approved by the review boards of all participating institutions. Informed written consent was received from all participants.

## Conflicts of Interest

L.S. acts as a consultant for Shenzhen MirrorEgo Technology Co. Ltd.

## Peer Review

The peer review history for this article is available at https://publons.com/publon/10.1002/brb3.70893.

## Supporting information




**Supplementary Tables**: brb370893‐sup‐0001‐TableS1‐S3.docx

## Data Availability

The data that support the findings of this study are available on request from the corresponding author. The data are not publicly available due to privacy or ethical restrictions.
