# Correction to “Baseline Impaired Insight Predicts Longitudinal Brain Atrophy in Alzheimer's Disease and Related Cognitive States: A 30‐Month Cohort Study From the ADNI Dataset”

**DOI:** 10.1002/brb3.71028

**Published:** 2025-10-31

Wilkins, J., C. Muñoz‐Neira, and L. Su. 2025. “Baseline Impaired Insight Predicts Longitudinal Brain Atrophy in Alzheimer's Disease and Related Cognitive States: A 30‐Month Cohort Study From the ADNI Dataset.” *Brain and Behavior* 15, no. 10: e70893. https://doi.org/10.1002/brb3.70893

In the originally published version, there was an error in the rendering of the second author's name, affiliations, and email address. The correct information is as follows:

Carlos Muñoz‐Neira

Sheffield Institute for Translational Neuroscience (SITraN), Division of Neuroscience, School of Medicine and Population Health, Faculty of Health, University of Sheffield, Sheffield, UK

Insigneo Institute for In Silico Medicine, School of Medicine and Population Health, University of Sheffield, Sheffield, UK

Correspondance email: carlos.munozn@uoh.cl

The online version has been corrected accordingly.

We apologize for this error.

