# Supplementary material for: Baseline Impaired Insight Predicts Longitudinal Brain Atrophy in Alzheimer's Disease and Related Cognitive States: A 30‐Month Cohort Study From the ADNI Dataset
Source: Brain Behav. 2025 Sep 30;15(10):e70893. doi: 10.1002/brb3.70893 (PMC12480922; doi:10.1002/brb3.70893)
Supplement: Supplementary file 1 — Supplementary Tables: brb370893‐sup‐0001‐TableS1‐S3.docx [file BRB3-15-e70893-s001.docx]

| **Supplementary table 1:** ANOVA of Overall Insight between diagnostic groups with Tukey Post Hoc comparisons. | | | | | | | | | | | |
| --- | --- | --- | --- | --- | --- | --- | --- | --- | --- | --- | --- |
| **Cases** | | **Sum of Squares** | | **df** | | **Mean Square** | | **F** | | **p** | |
| Diagnostic Group |  | 64.619 |  | 4 |  | 16.155 |  | 48.795 |  | < .001 |  |
| Residuals |  | 260.221 |  | 786 |  | 0.331 |  |  |  |  |  |
|  | | | | | | | | | | | |
| *Note.*  Type III Sum of Squares | | | | | | | | | | | |
| **Post Hoc Comparisons – Diagnostic Group** | | | | | | | | | | | |
|  | |  | | **Mean Difference** | | **SE** | | **t** | | **p_tukey_** | |
| CN |  | SMC |  | 0.144 |  | 0.064 |  | 2.244 |  | 0.165 |  |
|  |  | EMCI |  | 0.039 |  | 0.057 |  | 0.674 |  | 0.962 |  |
|  |  | LMCI |  | -0.236 |  | 0.064 |  | -3.667 |  | 0.002 | ** |
|  |  | AD |  | -0.889 |  | 0.079 |  | -11.18 |  | < .001 | *** |
| SMC |  | EMCI |  | -0.105 |  | 0.06 |  | -1.765 |  | 0.395 |  |
|  |  | LMCI |  | -0.38 |  | 0.067 |  | -5.697 |  | < .001 | *** |
|  |  | AD |  | -1.032 |  | 0.081 |  | -12.688 |  | < .001 | *** |
| EMCI |  | LMCI |  | -0.274 |  | 0.06 |  | -4.578 |  | < .001 | *** |
|  |  | AD |  | -0.927 |  | 0.076 |  | -12.203 |  | < .001 | *** |
| LMCI |  | AD |  | -0.653 |  | 0.082 |  | -8.002 |  | < .001 | *** |
|  | | | | | | | | | | | |
| ** p < .01, *** p < .001 | | | | | | | | | | | |
| *Note.*  P-value adjusted for comparing a family of 5 | | | | | | | | | | | |

| **Supplementary Table 2:** ANOVA of Whole Brain Annualised Change between diagnostic groups with Tukey Post Hoc comparisons. | | | | | | | | | | | |
| --- | --- | --- | --- | --- | --- | --- | --- | --- | --- | --- | --- |
| **Cases** | | **Sum of Squares** | | **df** | | **Mean Square** | | **F** | | **p** | |
| Diagnostic Group |  | 67.349 |  | 4 |  | 16.837 |  | 18.471 |  | < .001 |  |
| Residuals |  | 719.208 |  | 789 |  | 0.912 |  |  |  |  |  |
|  | | | | | | | | | | | |
| *Note.*  Type III Sum of Squares | | | | | | | | | | | |
| **Post Hoc Comparisons – Whole Brain Annualised Change** | | | | | | | | | | | |
|  | |  | | **Mean Difference** | | **SE** | | **t** | | **p_tukey_** | |
| CN |  | SMC |  | -0.094 |  | 0.106 |  | -0.887 |  | 0.902 |  |
|  |  | EMCI |  | 0.237 |  | 0.094 |  | 2.511 |  | 0.089 |  |
|  |  | LMCI |  | 0.322 |  | 0.106 |  | 3.021 |  | 0.022 | * |
|  |  | AD |  | 0.973 |  | 0.132 |  | 7.393 |  | < .001 | *** |
| SMC |  | EMCI |  | 0.331 |  | 0.099 |  | 3.344 |  | 0.008 | ** |
|  |  | LMCI |  | 0.416 |  | 0.111 |  | 3.759 |  | 0.002 | ** |
|  |  | AD |  | 1.067 |  | 0.135 |  | 7.906 |  | < .001 | *** |
| EMCI |  | LMCI |  | 0.085 |  | 0.099 |  | 0.853 |  | 0.914 |  |
|  |  | AD |  | 0.736 |  | 0.126 |  | 5.845 |  | < .001 | *** |
| LMCI |  | AD |  | 0.652 |  | 0.135 |  | 4.815 |  | < .001 | *** |
|  | | | | | | | | | | | |
| *Note.*  P-value adjusted for comparing a family of 5 | | | | | | | | | | | |
| * p < .05, ** p < .01, *** p < .001 | | | | | | | | | | | |

Supplementary Table 3 – STROBE Checklist for cohort studies

| Section/Topic | Item No. | Recommendation | Where it is found in the manuscript |
| --- | --- | --- | --- |
| Title and abstract | 1a | Indicate the study’s design with a commonly used term in the title or the abstract. | The title contains "30-month cohort study" and the abstract contains "a cohort of subjects". |
|  | 1b | Provide in the abstract an informative and balanced summary of what was done and what was found. | The "Abstract" section provides a summary of the background, objective, methods, results, and discussion. |
| Introduction | 2 | Background/rationale: Explain the scientific background and rationale for the investigation being reported. | The "Introduction" section explains the scientific background of dementia and the importance of studying insight in Alzheimer's disease. |
|  | 3 | Objectives: State specific objectives, including any prespecified hypotheses. | The "Objective" section states the specific objectives of the study. |
| Methods | 4 | Study design: Present key elements of study design early in the paper. | The "Methods" section details the study design. |
|  | 5 | Setting: Describe the setting, locations, and relevant dates, including periods of recruitment, exposure, follow-up, and data collection. | The study was conducted using data from the Alzheimer’s Disease Neuroimaging Initiative (ADNI) database, and the follow-up period was 30 months. The "Data collection" and "Subjects" subsections provide more details. |
|  | 6a | Participants: Give the eligibility criteria, and the sources and methods of selection of participants. Describe methods of follow-up. | The "Subjects" subsection of the "Methods" section details the eligibility criteria for participants and the source of the data, the ADNI database. |
|  | 6b | For matched studies, give matching criteria and number of exposed and unexposed. | The study was not a matched study. |
|  | 7 | Variables: Clearly define all outcomes, exposures, predictors, potential confounders, and effect modifiers. Give diagnostic criteria, if applicable. | The "Insight Assessment" and "MRI acquisition and analysis" subsections define the outcomes and exposures. The "Confounding variables" subsection defines the confounders. The "Subjects" subsection mentions that subgroups were categorized according to predefined criteria. |
|  | 8 | Data sources/measurement: For each variable of interest, give sources of data and details of methods of assessment (measurement). Describe comparability of assessment methods if there is more than one group. | The "Data collection" subsection notes that data was obtained from the ADNI database. The "Insight Assessment" and "MRI acquisition and analysis" subsections detail the methods of assessment. |
|  | 9 | Bias: Describe any efforts to address potential sources of bias. | The "Annualised brain atrophy rate" subsection describes the removal of outliers. The "Confounding variables" subsection describes how potential confounders were controlled. The "Limitations" section also discusses potential biases. |
|  | 10 | Study size: Explain how the study size was arrived at. | The "Subjects" subsection mentions that filtering the database resulted in 817 eligible participants. The "Participant demographics" subsection states that after removing 23 participants due to outlier ICV slope distribution, the final sample size was 794. |
|  | 11 | Quantitative variables: Explain how quantitative variables were handled in the analyses. If applicable, describe which groupings were chosen and why. | The "Statistical procedures" subsection explains how quantitative variables were handled, including the use of Shapiro-Wilk tests to assess normality. The "Insight Assessment" and "Annualised brain atrophy rate" subsections describe how scores and rates were calculated. |
|  | 12a | Statistical methods: Describe all statistical methods, including those used to control for confounding. | The "Statistical procedures" subsection describes the statistical methods used, including the use of JASP, FDR correction, Chi-squared, and Shapiro-Wilk tests. The "Confounding variables" and "Main results" sections describe how confounding variables were controlled. |
|  | 12b | Describe any methods used to examine subgroups and interactions. | The "Statistical procedures" subsection describes the comparison of variables between diagnostic groups. |
|  | 12c | Explain how missing data were addressed. | The "Subjects" subsection describes the eligibility criteria which required participants to have certain data, addressing missing data by exclusion. |
|  | 12d | If applicable, explain how loss to follow-up was addressed. | The "Subjects" subsection mentions the cutoff for follow-up visits was month 30 to ensure comparable data points between groups. |
|  | 12e | Describe any sensitivity analyses. | Not applicable. |
| Results | 13a | Participants: Report numbers of individuals at each stage of the study—e.g., numbers potentially eligible, examined for eligibility, confirmed eligible, included in the study, completing follow-up, and analyzed. | The "Participant demographics" subsection details the total sample size and the number of participants in each diagnostic group. |
|  | 13b | Give reasons for non-participation at each stage. | The "Participant demographics" subsection states that 23 participants were excluded for being extreme outliers in the ICV slope distribution. |
|  | 13c | Consider use of a flow diagram. | A flow diagram was not used. |
|  | 14a | Descriptive data: Give characteristics of study participants (e.g., demographics, clinical factors, comorbidities) and information on exposures and potential confounders. | The "Participant demographics" subsection and Table 1 provide demographic information (age, YoE, gender, MoCA, NoV) for the study population. |
|  | 14b | Indicate number of participants with missing data for each variable of interest. | The document does not provide a breakdown of missing data for each variable as a prescreening criteria was used of the ADNI dataset to ensure all participants had all varaible information. |
|  | 14c | Summarise follow-up time (e.g., average and total amount). | The study was a 30-month cohort study. Table 1 shows the mean number of clinic visits per group. |
|  | 15 | Outcome data: Report the number of outcome events or summary measures of risk over time. | The "Brain atrophy rates" subsection and Table 2 report the mean annualised percentage change in brain volume for each diagnostic group and brain region. |
|  | 16a | Main results: Give unadjusted and confounder-adjusted estimates and their precision (e.g., 95% confidence interval). | The "Results" section reports confounder-adjusted Spearman's rank correlation coefficients (rho) and p-values. |
|  | 16b | If applicable, report category boundaries when continuous variables were categorized. | The "Insight Assessment" subsection explains that insight scores were categorized as positive (overestimation) or negative (underestimation). |
|  | 16c | If relevant, consider translating estimates of relative risk into absolute risk for a meaningful time period. | Not applicable. |
|  | 17 | Other analyses: Report other analyses done—e.g., analyses of subgroups and interactions, and sensitivity analyses. | The "Insight levels by diagnostic group" subsection and Figure 2 show the distribution of insight scores by diagnostic group. The "Results" section also details correlations between subdomains of insight and regional brain changes in Table 3. |
| Discussion | 18 | Key results: Summarize key results with reference to study objectives. | The "Discussion" section summarizes the key findings and references the hypothesis. The "Abstract" and "What this study adds" sections also summarize the key results. |
|  | 19 | Limitations: Discuss limitations of the study, taking into account sources of potential bias or imprecision. Discuss both direction and magnitude of any potential bias. | The "Limitations and Future Directions" subsection discusses limitations such as the use of multiple ADNI studies, the assessment of insight only at baseline, and potential selection bias. It also notes that the associations were weak, suggesting limited predictive ability. |
|  | 20 | Interpretation: Give a cautious overall interpretation of results considering objectives, limitations, multiple analyses, results from similar studies, and other relevant evidence. | The "Discussion" section provides a cautious interpretation of the results, considering the study's findings in the context of existing literature. It discusses the implications of the findings and the need for further research. |
|  | 21 | Generalisability: Discuss the generalisability (external validity) of the study results. | The "Discussion" section highlights that the study's findings may stem further research into non-invasive predictors of dementia and their clinical utility. |
| Other information | 22 | Funding: Give the source of funding and the role of the funders for the present study and, if applicable, for the original study on which the present article is based. | The "Acknowledgements" and "Funding" sections detail the funding sources for the study and the roles of the funders. |
